# The reliability of the ankle-brachial index in the Atherosclerosis Risk in Communities (ARIC) study and the NHLBI Family Heart Study (FHS)

**DOI:** 10.1186/1471-2261-6-7

**Published:** 2006-02-21

**Authors:** Beth D Weatherley, Lloyd E Chambless, Gerardo Heiss, Diane J Catellier, Curtis R Ellison

**Affiliations:** 1 Duke Clinical Research Institute, Duke University Medical Center, Durham, NC, USA; 2 Department of Biostatistics, School of Public Health, University of North Carolina, Chapel Hill, NC, USA; 3 Department of Epidemiology, School of Public Health, University of North Carolina, Chapel Hill, NC, USA; 4 Section of Preventive Medicine and Epidemiology, Boston University Medical Center, Boston, MA, USA

## Abstract

**Background:**

A low ankle-brachial index (ABI) is associated with increased risk of coronary heart disease, stroke, and death. Regression model parameter estimates may be biased due to measurement error when the ABI is included as a predictor in regression models, but may be corrected if the reliability coefficient, R, is known. The R for the ABI computed from DINAMAP™ readings of the ankle and brachial SBP is not known.

**Methods:**

A total of 119 participants in both the Atherosclerosis Risk in Communities (ARIC) study and the NHLBI Family Heart Study (FHS) had repeat ABIs taken within 1 year, using a common protocol, automated oscillometric blood pressure measurement devices, and technician pool.

**Results:**

The estimated reliability coefficient for the ankle systolic blood pressure (SBP) was 0.68 (95% CI: 0.57, 0.77) and for the brachial SBP was 0.74 (95% CI: 0.62, 0.83). The reliability for the ABI based on single ankle and arm SBPs was 0.61 (95% CI: 0.50, 0.70) and the reliability of the ABI computed as the ratio of the average of two ankle SBPs to two arm SBPs was estimated from simulated data as 0.70.

**Conclusion:**

These reliability estimates may be used to obtain unbiased parameter estimates if the ABI is included in regression models. Our results suggest the need for repeated measures of the ABI in clinical practice, preferably within visits and also over time, before diagnosing peripheral artery disease and before making therapeutic decisions.

## Background

The ratio of the ankle to the brachial systolic blood pressure (SBP), the ankle-brachial index (ABI), is an indicator of atherosclerotic vascular disease in the lower extremities and a simple, non-invasive measure of subclinical atherosclerosis [1-9]. The ABI has been shown in cross-sectional studies to be associated with cardiovascular disease (CVD) risk factors including smoking [10-17] diabetes [10-12,18,19], total cholesterol [10,12,13,20,21] hypertension [10,12,13] and low birth weight [14]. The ABI is associated with other subclinical [10,22-25] and clinical [10,12,13,23,25-28] CVD manifestations. Prospective studies have found that those with ABI-defined lower extremity arterial disease (LEAD) are approximately 1.5 to 2 times more likely to have a clinical CVD event [27,29-31]. The ABI has a graded, inverse association with mortality [32,33].

Regression parameters may be biased when predictors are measured with error [34,35]. The parameter estimate for a regression model with a single explanatory variable measured with error will be biased towards the null by a multiplicative factor *R*, the reliability coefficient. Parameter estimates for additional covariates included in the model may be biased in any direction. Unbiased parameter estimates may be obtained using Stein estimators of true values [36] or other techniques if *R *is known [34,35].

To measure ankle SBP, Doppler ultrasound devices have been used for many years to detect blood flow distal to an occluding cuff. Many epidemiologic CVD studies have included an ABI measured using either Doppler at the ankle and sphygmomanometry at the arm, or Doppler at both the ankle and the arm. In order to reduce interobserver variation, several studies including the Atherosclerosis Risk in Communities (ARIC) study [37] and the NHLBI Family Heart Study (FHS) [38] have employed an automated, oscillometric device to measure ankle and brachial SBPs. Limited data regarding the repeatability of ankle and arm SBPs measured with oscillometric devices are available, but the repeatability of the ABI measured with the widely-used DINAMAP™ has not been published to our knowledge.

DINAMAP™ ankle and brachial SBPs were taken during ultrasound exams using the same protocol, equipment, and pool of sonographers in the ARIC study and the FHS. Using repeat measures of the ABI taken within a year apart for each of these studies, we estimate variance components and reliability coefficients for ankle and brachial SBPs and for the ABI. We also examined the effect of participant characteristics on the ABI reliability.

## Methods

### Study population

The ARIC study cohort comprises 15,792 members aged 45–64 years of randomly selected households in four United States communities: Forsyth County, North Carolina; northwest suburbs of Minneapolis, Minnesota; Washington County, Maryland; and African American residents of Jackson, Mississippi [37,39]. ARIC participants were examined at baseline between December 1986 and January 1990 and were then examined every 3 years after their baseline examination. Institutional review board (IRB) approval for each examination cycle and for annual follow-up were obtained by each participating field center and the coordinating center. Informed consent itemized the procedures consented to and any restrictions to the use of biospecimen. The FHS population comprises 588 randomly-selected individuals (probands) and their families and 657 families with high coronary heart disease (CHD) risk scores from three ongoing epidemiologic studies in four communities: the Forsyth County, North Carolina and Minneapolis, Minnesota cohorts of the ARIC study; the Framingham Heart Study in Framingham, Massachusetts; and the Health Family Tree Program in Salt Lake City, Utah [38]. FHS participants were examined in 1994–1995. IRB approval for the examination and a follow-up were obtained by the participating field centers and the coordinating center. Informed consent itemized the procedures consented to and as well as restrictions to the use of biospecimens.

Seven hundred ten ARIC study participants and their families participated in the FHS clinic examination: 267 randomly-selected families, 340 families with high family risk scores, and 103 black participants at Forsyth [38]. One member of each selected ARIC household was designated as proband; 585 total ARIC probands were examined in Phase II of the FHS, of whom 577 had an ARIC 6- or 9-year follow-up visit when the ABI was to be measured.

Replicate ankle-brachial index measures taken for each of the two studies were available for 335 ARIC probands, 120 of which were taken within 365 days. The ARIC ABI measure closest to the FHS exam was chosen; 48 participants had 3 measures, but in all cases the second and third measures were > 365 days apart. One participant who had a difference between ABI measures that was more than 3 standard deviations (SDs) from the mean pair difference was excluded. Of the remaining 119 participants, 25 (21 percent) had their first measure in the FHS and the second at ARIC visit 3 and 22 (18 percent) had the second at ARIC visit 4; 72 (61 percent) had the first ABI measure at ARIC visit 3 and the second at the FHS exam. Measures were taken an average of 228 days apart.

### Ankle-brachial index measurement

Ankle and brachial blood pressures were obtained immediately prior to the ultrasound examinations by the same pool of 11 sonographers in both the FHS and ARIC follow-up visit clinic exams, using the same protocol and equipment [40,41]. With the participant resting in the supine position, the cuff was placed using a contour wrapping technique over the posterior tibial artery of one ankle [42], selected based on the date. The cuff was then placed over the brachial artery of the right arm, if anatomy permitted. Blood pressures were taken with a DINAMAP™ 1846 SX automated oscillometric device (Critikon, Inc., Tampa, FL). None of the 577 subjects in both studies had a recorded blood pressure outside the device's detection limits, <30 or >245 mmHg [43]; one subject was excluded who had one ABI where the ankle systolic blood pressure (SBP) was ≥ 75 mmHg more than the brachial SBP, a criterion employed to exclude non-compressible arteries [44]. The ABI was computed as the ratio of the ankle SBP to the brachial SBP.

At the ARIC baseline survey, the ankle SBP was measured with the participant prone before and after ultrasound scanning of the popliteal artery, then brachial SBPs were measured supine every 5 minutes during the carotid ultrasound. The ABI was computed as the ratio of the average of the last two available ankle SBPs to the average of the first two brachial SBPs.

### Covariates

In the ARIC study and the FHS, hypertension was defined as a sitting SBP > 140 mmHg, diastolic blood pressure > 90 mmHg, or use of antihypertensive medication within 2 weeks prior to the examination. Diabetes was defined as a fasting glucose level ≥ 7.8 mmol/L (140 mg/dL), a non-fasting level ≥ 11.1 mmol/L (200 mg/dL), self-reported history of diabetes, or the use of hypoglycemic agents. In ARIC, prevalent CHD was defined as evidence of a prior myocardial infarction on a 12-lead ECG, or self-reported history of a physician-diagnosed heart attack, coronary bypass surgery, or coronary angioplasty. In the FHS self-reported, physician diagnosed coronary heart disease was confirmed by review of hospital records.

### Statistical analysis

For all statistical tests, two-sided *p *values < 0.05 were considered statistically significant. Characteristics of the ABI reliability study subjects were compared to characteristics of the ARIC source population at visits 1 and 3 using Kruskal-Wallis two-sample tests for continuous variables and Wilcoxon tests for categorical variables.

Each of the two observed values was assumed to represent the sum of the true value and random within-person variation [34]. Within-person variation may be due to measurement error and physiologic variation. We assumed that the within-person variation is independent of the value's magnitude and that the within-person variation for each of the two values are independent and identically distributed with mean zero and common variance. Estimates of the between-person and within-person variances were obtained with estimators derived using the method of moments; similar estimates were obtained using SAS PROC MIXED release 8.1 (SAS Institute, Inc., Cary, NC). The reliability coefficient, *R*, was computed as the ratio of the between-person variance to the total variance and can be interpreted as the proportion of the total variance not due to within-person variation, or as the correlation between measures made at repeat visits for an individual. The within-person coefficient of variation (CV) was computed as 100 times the square root of the within-person variance divided by the mean of the individual mean replicates. These methods were applied to ankle and brachial SBP measurements as well as to ABI. In addition, the multivariate generalization of this method was applied to produce the multivariate error covariance matrices and between-person covariance matrices for ankle and arm SBP and the component-wise ratios of the error and between-person covariance to total covariance. (The diagonal elements of this matrix of ratios are the reliability coefficients for ankle and arm SBP).

Because the arm SBPs and ABIs were not normally distributed, confidence intervals for the estimates of variance components and reliability coefficients were derived from 300 bootstrap samples [45]. We compared variance components, reliability coefficients, and CVs by levels of important covariates, including the FHS sampling group. Analyses did not account for the FHS sampling scheme. To test subgroup differences, we constructed bootstrapped 95 percent confidence intervals for the differences; intervals not including zero were deemed statistically significant.

To examine the assumption of independence of the within-person variation from the ABI level, we compared the estimated square root of the within-person variance (i.e., the within-person SD) for those whose first of the two repeat ABIs was at or below with those whose first ABI was above the median ABI in the 119 reliability study subjects. We also examined plots of the pair differences versus the pair means for the ankle SBP, arm SBP, and ABI [46].

The ABI measured at the ARIC baseline survey is of interest as a predictor of subsequent events. Because repeat ankle and arm SBPs are available for the baseline survey, the ABI is usually computed as the ratio of the average of two ankle SBPs (in the same ankle) to the average of two arm SBPs (in the right arm). We estimated the within-visit multivariate error and total covariance matrices using repeat ankle and arm SBP measures taken at the ARIC baseline survey. The ratios of within-visit multivariate error to total covariance matrices from the baseline survey data were multiplied times the total variance in the ARIC/FHS repeat data to partition the non-between person variance found there into between-visit and within-visit components. We simulated a two-measures-per-visit repeatability dataset very much like the actual repeatability dataset by taking each subject's mean of the two actual repeated BP measures as the "true" BP values, and adding independent random "between-visit" and "within-visit" Gaussian measurement error, of size to make these two variance components the same proportion of the total variance as in the results of the repeatability study and the baseline survey within-visit repeatability analysis. With the simulated data, the reliability coefficient for the ABI was computed as a ratio of means, along with the reliability coefficient using just the first measure at each visit. This was repeated 1000 times and the reliability coefficients averaged.

## Results

Of the 119 reliability study subjects, 70 (58.8 percent) were women, 100 (84.0 percent) were white, and 77 (64.7 percent) were from the Forsyth County field center. At the time of the first ABI measure, the mean age was 61.3 years, mean weight 75.7 kg, mean body mass index 28.0 kg/m^2^, and mean ABI 1.16; 13 (10.9 percent) had diabetes mellitus, 43 (36.1 percent) had hypertension, and 31 (26.0 percent) had coronary heart disease. Characteristics of this group were similar to those of the ARIC cohort in Minneapolis and Forsyth County at the time of the ARIC baseline survey and at visit 3, except with respect to the distribution of characteristics related to the FHS sampling procedures: race/ethnicity, field center, and CHD prevalence (Table 1).

**Table 1:** Characteristics of ABI reliability study subjects compared with the ARIC study cohort

	**ARIC baseline survey**		**ARIC visit 3**	
	
**Characteristic**	**ARIC Minneapolis and Forsyth County** ** (N = 8044)**	**ABI reliability study** ** (N = 119)**	**ARIC Minneapolis and Forsyth County** ** (N = 6839)**^†^	**ABI reliability study** ** (N = 119)**
Age, y*	54.2 (5.79)	55.0 (5.68)	60.2 (5.73)	61.0 (5.67)
Female, %	52.8	58.8	53.4	58.8
Race/ethnicity, %				
White	93.3	84.0§	94.5	84.0§
African American	6.3	16.0	5.1	16.0
American or Alaskan Indian	0.1	0	0.1	0
Asian or Pacific Islander	0.3	0	0.3	0
Field center, %				
Forsyth County, NC	50.2	64.7^‡^	48.9	64.7§
Minneapolis, Minn	49.8	35.3	51.1	35.3
Weight, kg*	76.0 (16.21)	75.7 (15.63)	78.1 (16.79)	78.4 (17.12)
Body mass index, kg/m^2^*	26.7 (4.73)	27.0 (4.78)	27.6 (5.03)	28.1 (5.34)
Diabetes, %	6.7	7.6	12.0	11.8
Smoking status, %				
Current	26.8	23.5	18.2	19.3
Former	35.7	33.6	44.8	42.9
Never	37.5	42.9	37.0	37.8
Hypertension, %	26.6	26.9	34.4	35.3
Coronary heart disease, %	4.6	18.0§	6.8	23.7§
Ankle brachial index*	1.14 (0.130)	1.13 (0.137)	1.19 (0.150)	1.17 (0.150)

*Numbers presented are mean (SD).

^† ^ARIC participants with a visit 3 (6-year follow-up); for the ABI, N = 2295.

^‡ ^0.001 ≤ *p *< 0.01 for chi-square test comparing sample to source study population.

§ *p *< 0.001

The estimated reliability coefficient for the ankle SBP was 0.682 (95 percent confidence interval (CI): 0.570, 0.772), for the arm SBP was 0.736 (95 percent CI: 0.622, 0.827), and for the ABI was 0.612 (95 percent CI: 0.505, 0.699) (Table 2). These values are very close to estimated Pearson correlation coefficients of 0.68, 0.74, and 0.62, respectively, which have no underlying measurement error model assumptions. Arm SBP measures appear to have been more repeatable than ankle SBPs, as reflected by a lower within-person SD (10.8 v. 14.6 mmHg), a lower within-person CV (8.6 v. 10.0 percent), and a higher reliability coefficient (0.736 v. 0.682). Exclusion of 4 data pairs with either ABI measurement > 1.5 had little effect on variance estimates.

**Table 2:** Variation in DINAMAP™ measurements of arm and ankle systolic blood pressure and the ankle-brachial index

**Estimate**	**Ankle SBP**	**Arm SBP**	**ABI**
Mean (SD) of pair means, mmHg*	146.2 (23.76)	125.2 (19.57)	1.178 (0.1522)
Mean (SD) of pair differences, mmHg*	-3.1 (20.67)	0.9 (15.24)	-0.031 (0.1494)^‡^
Square root of variance components, mmHg*			
Between-person^†^	21.4 (17.6,25.1)	18.0 (14.5, 1.3)	0.133 (0.112,0.150)
Within-person^†^	14.6 (12.7,16.5)	10.8 (8.9,12.4)	0.106 (0.091,0.121)
Total^†^	25.9 (22.8,28.7)	21.0 (18.3, 3.7)	0.170 (0.151,0.185)
Reliability coefficient^†^	0.682 (0.570, 0.772)	0.736 (0.622, 0.827)	0.612 (0.505, 0.699)
Within-person coefficient of variation, %^†^	10.0 (8.6, 11.2)	8.6 (7.1, 9.9)	9.0 (7.7, 10.2)
Pearson correlation coefficient	0.68	0.74	0.62

*Systolic blood pressures (SBPs) in mmHg; the ankle-brachial index (ABI) is without units.

Difference represents first minus second measure.

^†^Estimate (95% confidence interval).

^‡ ^*p *< .05 for t-test of zero mean. Adjustment for the fixed effect of time changed very little the estimates of variance components.

The ankle SBP increased an average of 3.1 mmHg between the first and second measures (*p *= 0.11), which resulted in a statistically significant average increase of 0.031 in the ABI (*p *= 0.02). Adjustment for the fixed effect of time (first v. second measure) in an analysis of variance model changed the variance component estimates very little. FHS ankle SBP measures were an average of 3.2 mmHg higher than ARIC measures (*p *= 0.10). ABI measures in the FHS were 0.029 higher, on average, than ARIC measures (*p *= 0.03); controlling for the fixed effect of time reduced the study difference to 0.024 (*p *= 0.09). No significant effects of time or study were apparent for arm SBP.

The estimated ABI variance components differed in some subgroups (Table 3). The within-person SD was statistically significantly smaller at the Minneapolis than at the Forsyth County field center (0.086 versus 0.116). The within-person SD was smaller in those with a BMI ≤ the median of 27 kg/m^2 ^than in those with a BMI > 27 kg/m^2 ^(0.083 versus 0.126), resulting in a significantly greater reliability coefficient in those with a lower compared to those with a higher BMI (0.741 versus 0.495). Those whose same ankles were measured had a statistically significantly lower reliability coefficient (*R *= 0.486) than those with different ankles measured (*R *= 0.706). There were too few African American subjects, subjects with diabetes, and subjects who initiated or discontinued hypertension treatment to test for subgroup differences. Within-person SDs and reliability coefficients did not differ significantly by age ≤ or > 62 years, gender, hypertension, or by whether the time between measures was greater or less than the median interval of 240 days. Except for the estimated total variance, estimates did not differ significantly between those in the high family risk score and the random sample FHS sampling groups; the proportion of reliability study subjects in the African American sampling group was too small to test for differences.

**Table 3:** Variation in DINAMAP™ measurements of the ankle-brachial index by subgroup defined at the first measurement

		**Square root of variance components**				
				
Subgroup	**N**	**Between-person**	**Within-person**	**Total**	**Reliability Coefficient**	**Within-person CV, %**
Overall	119	0.133	0.106	0.170	0.612	9.0
						
Age, y						
≤ 62	66	0.124	0.104	0.162	0.587	8.9
> 62	53	0.144	0.107	0.180	0.643	9.1
						
Race/ethnicity						
African American^†^	19	0.098	0.079	0.125	0.606	6.8
White	100	0.139	0.110	0.177	0.614	9.3
						
Gender						
Male	49	0.140	0.102	0.173	0.653	8.4
Female	70	0.122	0.109	0.163	0.555	9.5
						
Field center						
Forsyth County, NC	77	0.127	0.116*	0.172	0.545	9.9*
Minneapolis, Minn	42	0.142	0.086*	0.166	0.732	7.2*
						
Body mass index, kg/m^2^						
≤ 27	61	0.141	0.083*	0.164	0.741*	7.1*
> 27	58	0.124	0.126*	0.178	0.495*	10.7*
						
Diabetes						
No	106	0.134	0.099	0.167	0.646	8.4
Yes^†^	13	0.112	0.150	0.187	0.358	13.3
						
Hypertension						
No	76	0.128	0.100	0.163	0.622	8.3
Yes	43	0.134	0.116	0.177	0.574	10.1
						
Hypertension rx switch^‡^						
No	101	0.136	0.104	0.171	0.630	8.8
Yes^†^	18	0.114	0.115	0.162	0.498	9.9
						
Coronary heart disease						
No	88	0.129	0.098	0.162	0.638	8.3
Yes	31	0.148	0.116	0.188	0.619	9.9
						
Legs						
Same	51	0.115	0.118	0.165	0.486*	10.0
Different	68	0.146	0.094	0.174	0.706*	8.6
						
Days between measures						
≤ 240	60	0.131	0.107	0.169	0.602	9.1
> 240	59	0.136	0.103	0.171	0.633	8.7
						
FHS sampling group						
African American^†^	19	0.098	0.079	0.125	0.606	6.8
High family risk score	68	0.148	0.114	0.187*	0.629	9.7
Random sample	32	0.117	0.099	0.153*	0.581	8.2

*p < 0.05 for the difference between the two subgroups (the 95% bootstrapped confidence interval for the difference did not include zero).

^†^Proportion too small to perform statistical tests of differences.

^‡^"No" includes those who were either taking or not taking hypertension medication at the time of both ABI measures; "yes" includes those who were taking hypertension medication at the time of one ABI measure and not taking hypertension medication at the time of the other ABI measure.

**Figure 1:**
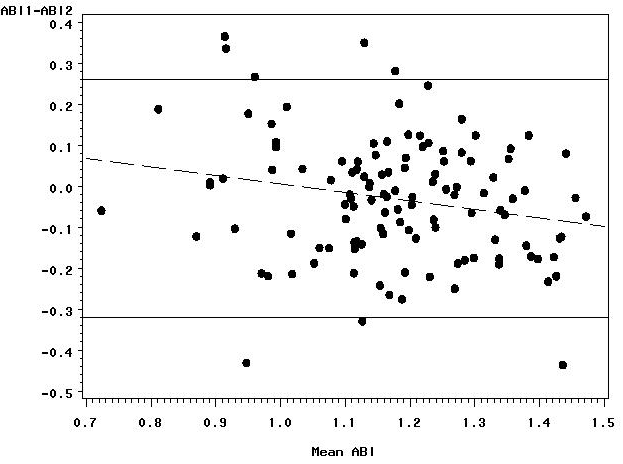
**Bland-Altman plot showing the reproducibility of the ankle-brachial index**. The differences between the first and second measurements are plotted against the mean of the two measures with 95% limits of agreement (solid lines) and regression line (dashed line).

**Figure 2:**
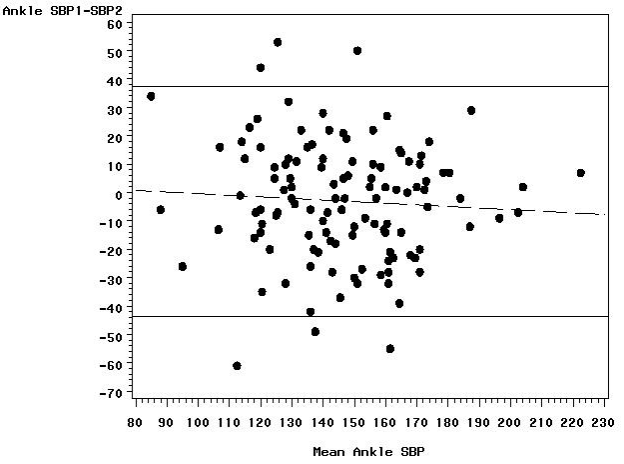
**Bland-Altman plot showing the reproducibility of the ankle systolic blood pressure**. The differences between the first and second measurements are plotted against the mean of the two measures with 95% limits of agreement (solid lines) and regression line (dashed line).

**Figure 3:**
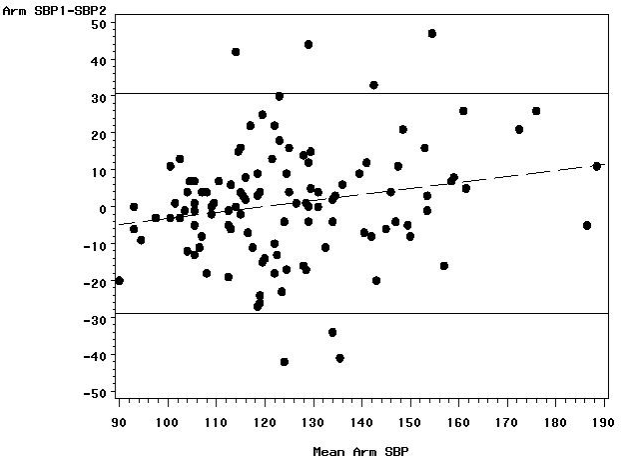
**Bland-Altman plot showing the reproducibility of the brachial systolic blood pressure**. The differences between the first and second measurements are plotted against the mean of the two measures with 95% limits of agreement (solid lines) and regression line (dashed line).

Within-person SD estimates did not differ significantly between those with an ABI ≤ 1.15 and those with an ABI > 1.15 (0.113 versus 0.097), the median ABI in the 119 reliability study subjects. A plot of the pair differences versus the pair means revealed no obvious dependence of the spread of the differences on the ABI level (Figures 1, 2, 3); however, the difference between the first and second measure decreased with increasing ABI level. The ankle SBP differences did not depend statistically significantly on ankle SBP level, but the arm pressure difference decreased with increasing arm SBP. The two ankle pressures differed by >10 mmHg in 73 (61.3 percent) subjects, by >20 mmHg in 38 (31.9 percent) subjects, by >30 mmHg in 15 (12.6 percent) subjects, and by >40 mmHg in 7 (5.9 percent) subjects. Arm pressures differed by >10 mmHg in 51 (42.9 percent) subjects, by >20 mmHg in 19 (16.0 percent) subjects, by >30 mmHg in 7 (5.9 percent) subjects, and by >40 mmHg in 5 (4.2 percent) subjects.

The multivariate within-person error covariance and true-to-total ratio covariance matrices for ankle and arm SBPs estimated from the reliability study are given in Table 4. The non-zero ankle-arm covariance suggests that the ankle and arm SBP within-person errors were correlated (ρ = 0.56). The diagonal elements of the true-to-total component-wise ratio covariance matrix represent the scalar reliability coefficients.

**Table 4:** Multivariate  between-visit covariance matrices for ankle and arm systolic blood pressures estimated in reliability study subjects

	Ankle SBP	Arm SBP
Within-person covariance		
Ankle SBP	213.6	88.3
Arm SBP	88.3	116.2
		
True/total ratio matrix		
Ankle SBP	0.682	0.741
Arm SBP	0.741	0.736

The multivariate within-person (between-visit) error covariance and true-to-total component-wise ratio covariance matrices estimated from the ARIC baseline survey are given in Table 5. The within-visit ankle and arm errors were essentially uncorrelated (ρ = 0). The correlation between within-visit measures was high: 0.92 for ankle SBP and 0.90 for arm SBP.

**Table 5:** Multivariate within-visit covariance matrices for ankle and arm systolic blood pressures estimated in ARIC study participants

	Ankle SBP	Arm SBP
Within-person covariance		
Ankle SBP	49.8	-0.0159
Arm SBP	-0.0159	44.0
		
True/total ratio matrix		
Ankle SBP	0.922	1.000
Arm SBP	1.000	0.900

From ARIC baseline survey data, we also estimated the within-visit variance and reliability of repeat ABIs, arbitrarily computed as the ratio of the first ankle to first arm SBP and the ratio of the second ankle to second arm SBP. The within-visit error variance for the ABI was 0.00636, and the reliability coefficient was 0.681.

The ABI measured at the ARIC baseline survey, computed as the ratio of the average of two ankle SBPs to the average of two arm SBPs, is of interest as a predictor in regression models. Using the estimates of the total and within-visit errors (Tables 4 and 5, respectively), a dataset containing two ankle and two arm SBPs at each of two visits was simulated by assuming that the pair means for the reliability study represented the "true" values. For each of 1000 replications, the reliability coefficients were estimated for the ABI computed at each visit as the ratio of the averages of the within-visit values and the ABI computed as the ratio of the first measures, and then the mean reliability coefficients over the replications taken. The result for the reliability coefficient for single measures was 0.613, very near that from the actual reliability study, and was 0.704 for the reliability coefficient for the ratio of means.

## Discussion

The within-person variance estimated from our data indicates that the 95% confidence interval of a patient's ABI would be the ABI value ± 0.21 if based on a single measure of the ABI. Thus, our results suggest the need for repeated measures of the ABI in clinical practice, if at all possible within visits and also over time, before diagnosing peripheral artery disease and before making therapeutic decisions.

To our knowledge, few studies of the repeatability of ankle or arm pressures measured with the DINAMAP™ have been published, and the designs of the few published studies do not include these same sources of variability. No reliability coefficient has been published, of which we are aware, for the ABI measured using the DINAMAP™ at both the ankle and arm.

We have estimated within-person SDs of 14.6 mmHg for ankle SBP and 10.8 mmHg for brachial SBP, with reliability coefficients of 0.68 and 0.74, respectively, for DINAMAP™ measures taken up to 1 year apart in a sample of two middle-aged populations. Using a DINAMAP™ 1846SX and a contour wrap at the ankle, Mundt et al [42] found a within-person SD of 4.0 mmHg for ankle SBP and 2.5 mmHg for brachial SBP, with reliability coefficients of 0.94 and 0.92, respectively, for three repeat ankle and two repeat arm measures one minute apart by one of two technicians among 71 adult volunteers aged 23–67 years; the same ankle was used for all repeat measures. Ramanathan et al [47] who also used a contour wrap at the ankle but used a VitalCare DOX Model 506DXN automated oscillometric device, estimated inter-reading intraclass correlation coefficients (ICCs) of 0.83 for ankle SBP and 0.85 for arm SBP for 3 repeat measures taken 30 minutes apart by each of 2 investigators among 50 healthy volunteers with a median age of 23 years. De Graaff et al [48] using a DINAMAP™ Plus measured brachial SBPs twice by each of two observers at each of 2 visits one week apart in 54 vascular laboratory patients with a mean age of 66 years. Estimates of within- and between-day, and within- and between-week, ICCs are given, but no overall estimate is presented with which we can compare our estimate of reliability.

Using the contour wrapping technique, ankle SBP measured with the DINAMAP™ 1846SX were found to be about 3.0 mmHg lower, on average, than measures taken with Doppler ultrasound [42]. Estimates of reliability were comparable between the two methods, with a reliability coefficient of 0.92 for Doppler and 0.94 for DINAMAP™ measures. This study did not include variation due to different days or legs, however. Fowkes et al [49] studied the variability of Doppler ultrasound measures of ankle SBP, brachial SBP, and the ABI in 24 peripheral artery disease (PAD) patients and 11 volunteers without PAD aged 40–74 years. Two measures were taken in each leg and the right arm by each of two observers and then repeated two weeks later. Reliability coefficients can be computed from the results by summing all intraindividual variance components. Regrettably, components of variance <10 were not reported, but the range of possible values can be estimated assuming unreported estimates were either all 10 or all 0. For Doppler ankle SBP, *R *was between 0.78 and 0.90 for normal subjects, and 0.37 and 0.46 for diseased subjects. For Doppler arm SBP, *R *was between 0.87 and 0.91 for normal subjects, and was 0.68 for diseased subjects. For the ABI, *R *was between 0.40 to 0.42 for diseased subjects; the range of *R *computed from the reported between-subject variance for normal subjects was implausibly low. These values bracket those found in our reliability study for DINAMAP™ SBP measures.

The estimated reliability coefficient for the ABI, 0.61, is comparable to that for another measure of subclinical atherosclerosis in the ARIC study, carotid intima-medial thickness (IMT), but is lower than that found for other measures. A reliability coefficient of 0.60 was estimated for the mean carotid IMT, averaged over 3 arterial sites bilaterally, in 279 subjects who participated in both the ARIC study and the FHS and with carotid B-mode ultrasounds within a year apart (Diane Catellier, University of North Carolina, unpublished manuscript, 2000). A reliability coefficient of 0.67 was estimated for the mean carotid IMT measured at three visits, 7–14 days apart in 36 volunteers from each of the four ARIC field centers [50]; measures of arterial distensibility and arterial compliance had reliability coefficients of 0.67 and 0.77 [51]. Estimated reliability coefficients for the ARIC study are 0.94 for both total and HDL-cholesterol [52], and 0.72 for plasma fibrinogen [53].

The within-person variation in this reliability study includes variation due to within and between observer, day, and leg, and physiologic variation. Thus, the ABI reliability estimated from the reliability study data is lower than the within-visit reliability estimated from ARIC baseline survey data, because the reliability study's within-person variability includes both between-visit and within-visit variation. The between-visit variation includes variation due to different technicians, different machines, and within-person biologic variation.

The reliability of the ABI was lower than that of either of its components: the ankle SBP or arm SBP. The lower reliability of the ankle SBP compared with that of the arm SBP may be due to the greater difficulty in placing a cuff on the more conical extremity.

Estimates of within-person variation in this reliability study apply to measures using the methodology at ARIC visits 3 and 4 and in the FHS, and may not apply to other situations. Extreme care was taken in these studies to minimize measurement variation, including a detailed, common protocol and training and certification of technicians. Reliability is likely to be lower in patient care settings, where these methods are unlikely to be standardized. Different oscillometric devices may vary in the algorithms employed for determining systolic blood pressure [54] and the accuracy of different oscillometric devices relative to sphygmomanometry has been found to differ [55]; the repeatability of different devices may differ as well. In addition, the reliability coefficient depends on the total variance in the population and, therefore, the reliability estimated in this study may not apply to other populations. Assuming the same within-person variance, if the total variance is lower than in the reliability study, as it is in the ARIC baseline survey, then the reliability coefficient would be lower than that estimated.

The reliability study defined the ABI as a ratio of single blood pressure measurements. In the ARIC baseline survey, the ABI is defined using the mean of two ankle SBPs (in the same leg) and the mean of two arm SBPs (in the same arm), where the measures were taken approximately 5 minutes apart, and in the study of associations between the ABI and potential disease outcomes this ABI will be used. We have seen that the reliability coefficient for such an ABI is notably higher than that computed directly from the ARIC/FHS reliability study.

In clinical practice, repeated measures of the ABI should be made, preferably within visits and over time before diagnosing peripheral artery disease and before making therapeutic decisions. Based on the within-person variance estimated here, the 95% confidence interval (1.96 times the within-person standard deviation) for one person based on one measure of the ABI would be the ABI ± 0.21. In general, for the average of *n *measures, this interval becomes the ABI average ± 0.21/√*n*, so the interval for an ABI based on the average of two visits would be ABI ± 0.15, and average ABI ± 0.12 if based on 3 measures. The reliability of oscillometric measurement of the brachial SBP is not much greater than that of the ABI, and multiple measures of the brachial SBP are recommended before instituting hypertension treatment [56].

Most population-based epidemiologic studies have measured the ankle pressure in both legs, once [18] or twice over the posterior tibial [10,12,57] or both over this artery and the dorsalis pedis [58] on each ankle. The mean of the repeated measurements on each ankle [10,12,57] or the highest SBP for each ankle [58] was then used, and a single ABI for the participant was computed using the minimum of the ankle numerators of the two legs. ABI values based on different protocols likely are associated with different levels of variability, the most robust (although not necessarily the most sensitive to peripheral arterial disease), being those based on averaged repeated measures. The effect of the choice of measurement protocol on the ABI reliability would be important to know, but cannot be assessed in this study since the ABI was defined in ARIC using ankle pressure measured in one, randomly selected leg.

The limited number of reliability study subjects in certain subgroups limited our ability to test for subgroup differences. The significantly higher within-person SD at Forsyth might be because 7 technicians were employed at the Forsyth County field Center and 4 at Minneapolis. The Forsyth County field center had 25 percent African American subjects, while Minneapolis subjects were all white (*p *< 0.001); Forsyth also had a higher proportion of subjects with diabetes (14 percent v. 5 percent) than Minneapolis. The center difference might also reflect different proportions of subjects with higher within-person variation, although the difference in reliability coefficients between the two centers was not larger than chance alone would explain and small numbers in these subgroups prevent further analysis. Counter to intuition, the ABI had a lower reliability in those whose ABI was measured in the same leg than in those whose was measured in different legs; this may represent a chance finding in this small study. It would have been desirable to examine whether the reliability differed in subjects with and without ABI-defined LEAD (e.g., ABI < 0.9), but the limited sample size did not allow for this analysis.

Individuals are often categorized as having LEAD or not by categorizing the observed ABI as below or above a cutpoint, typically 0.9. This categorical variable is then included as a predictor in regression models. An upper limit of normal for the ABI also has been proposed [57] to assess arterial stiffness and medial arterial calcification. Categorization of a continuous variable measured with non-differential measurement error may result in differential misclassification when the probability of disease is related to the level of the continuous variable [59]. Resulting regression parameter estimates are biased, but methods that assume non-differential misclassification may not correctly adjust these estimates.

## Conclusion

The reliability of the ABI computed as the ratio of single ankle and arm oscillometric SBPs was found to be 0.61. The reliability of the ABI computed as the ratio of the average of two ankle SBPs to two arm SBPs was estimated from simulated data as 0.70. These reliability estimates may be used to obtain unbiased parameter estimates if the ABI is included in regression models. If ankle and arm SBP are included as separate predictors in a regression model, corrected parameter estimates may be obtained by taking the multivariate reliability into account.

## Competing interests

The author(s) declare that they have no competing interests.

## Authors' contributions

GH and BW conceived of and designed the study. BW and LC performed the statistical analyses. GH, LC, and BW interpreted the results. BW drafted the manuscript. All authors revised the manuscript for intellectual content, and read and approved the final manuscript.

## Pre-publication history

The pre-publication history for this paper can be accessed here:


